# Identification of Novel Compound Heterozygous Mutations in the *GAN* Gene of a Chinese Patient Diagnosed With Giant Axonal Neuropathy

**DOI:** 10.3389/fnins.2020.00085

**Published:** 2020-02-25

**Authors:** Xiaomin Xu, Xiaokai Yang, Zhongliang Su, Hai Wang, Xiaoqing Li, Congcong Sun, Wenhuan Wang, Yao Chen, Chenhui Zhang, Hongping Zhang, Fan Jin, Jiayong Zheng

**Affiliations:** ^1^Wenzhou People’s Hospital, Wenzhou Maternal and Child Health Care Hospital, The Third Clinical Institute Affiliated to Wenzhou Medical University, Wenzhou, China; ^2^Wenzhou City Key Laboratory of Gynecology and Obstetrics, Wenzhou, China; ^3^Hangzhou Fuyang Women and Children Hospital, Fuyang, China; ^4^Women’s Hospital, School of Medicine, Zhejiang University, Hangzhou, China

**Keywords:** giant axonal neuropathy, point mutation, compound heterozygosity, targeted next-generation sequencing, disease attributes

## Abstract

Giant axonal neuropathy (GAN) is a very rare autosomal recessive disorder characterized by abnormally large and dysfunctional neuronal axons. Mutations in the *GAN* gene have been identified as the cause of this disorder. In this report, we performed a detailed phenotypic assessment of a Chinese patient with GAN. An array-based exon capture test and targeted next-generation sequencing were used to detect the suspected mutation sites. Compound heterozygous mutations of p.S79L (c.236C > T) in the BTB domain and p.T489S (c.1466C > G) in the kelch domain were identified in the proband’s genome. S79L was a known mutation, and T489S was reported for the first time. The p.S79L and p.T489S were confirmed in the proband’s mother and father, respectively. Both mutations were located in highly conserved regions and affected the predicted protein crystal structures. The proband’s sural biopsy revealed the classical GAN phenotype of swollen axons filled with closely packed neurofilaments. The combined application of the next-generation sequencing platform and bioinformatics analyses was an effective method for diagnosing GAN. The novel compound mutations of S79L and T489S in the *GAN* gene were likely the cause of the patient’s GAN symptoms. Our findings enrich the spectrum of mutations associated with this rare type of axonopathy.

## Introduction

Giant axonal neuropathy (GAN; OMIM#256850) is a very rare autosomal recessive disorder caused by specific mutations in the *GAN* gene (Bomont et al., 2000). Since the first GAN report in 1972, more than 50 *GAN* gene mutations have been identified in 80 cases around the world (Normendez-Martinez et al., 2018). According to clinical symptoms, GAN can be divided into classical phenotype and mild phenotype; both shared the physiological features of dense nerve fiber accumulation. In addition, the classical clinical phenotype is characterized by severe peripheral neuropathy, kinky hair, early onset of cerebellar and pyramidal signs, and mental deterioration (Tazir et al., 2009; Vijaykumar et al., 2015). However, the mild clinical phenotype has a relatively slow onset and progression (Tazir et al., 2009; Koichihara et al., 2016).

Detecting *GAN* gene mutations is the current method used in diagnosing GAN (Normendez-Martinez et al., 2018). The *GAN* gene is located at 16q24.1 and encodes gigaxonin. Gigaxonin contains an N-terminus BTB domain, a C-terminus kelch domain, and a BACK domain between the N-terminal and C-terminal ends. The gigaxonin protein has been found to regulate microtubule-binding protein degradation (Bomont et al., 2000). The regulation of microtubule-binding protein degradation may be a common pathological target in neurodegenerative disorders that arise from alterations in the neurofilament network (Bomont et al., 2000). The *GAN* gene mutations, which result in changed gigaxonin structure and nerve signal transduction complications, are the cause of the pathophysiology observed in GAN (Israeli et al., 2016). In addition to the genetics studies, multiple functional studies expanded our knowledge of GAN. The regulation of intermediate filaments may be of importance in addition to the regulation of microtubule-associated proteins (Mahammad et al., 2013), and recent evidence shows that gigaxonin may regulate autophagy (Scrivo et al., 2019).

In this study, we report the clinical presentation and genetic profile of a Chinese patient with compound heterozygous mutations in the *GAN* gene.

### Case Presentation

The patient described in this report was a 7-year-old boy conceived by unrelated parents. His hair was thick and curly. At 3 years of age, he developed a gait abnormality, walked slowly, fell easily when he ran, and had outward facing toes on his right foot. When he was 5 years old, he developed a mild obstructive sleep apnea–hypopnea syndrome, and his right hip was dislocated. After he underwent surgery on his hip, he suffered from muscle weakness and could not walk until 1 year later. At 7 years of age, the patient had normal intelligence, and his cervical region muscle strength was observed at level IV. He experienced a slight difficulty when trying to turn his neck. His muscle strength was at level V in the upper limbs, level IV in the in the lower limbs, and level IV in the feet, which was determined by dorsal extension tests. He performed poorly in the finger-to-nose test, especially when he was using his left hand. During this test, he clumsily alternated between the right and left hand. His pallesthesia of the upper and lower limbs was lost at distal locations more than proximal locations. The tendon reflex was absent, and he was unable to stand on one leg. Upon examining the cranial and spinal magnetic resonance imaging (MRI), electroencephalogram, and electrocardiogram test results, no abnormal signs were detected. Notably, the electromyography exam revealed neuronal damage, mainly located at the axons of multiple peripheral nerves. A subsequent electrophysiological examination supported the same conclusion. In the right median nerve, the level of CMAP was 2.1 m/s (amplitude, 2.6 mV; level of DL, 6.8 ms). In the left ulnar nerve, the level of CMAP was 62.2 m/s (amplitude, 2.4 mV; level of DL, 6.6 ms). The level of SNAP was 59.9 m/s in the left peroneus nerve (amplitude, 4.2 mV; level of DL, 1.4 ms), and 58.0 m/s in the right peroneus nerve (amplitude, 3.1 mV; level of DL, 1.3 ms). We calculated the Charcot–Marie–Tooth neuropathy score (CMTNS), and the result is 15 (moderate). No distal levels of CMAP or amplitude decreases were distinct from motor neurons of the lower limbs, and no distal SNAP levels from the upper limb sensory and bilateral plantar nerves were recorded. There was no history of neuromuscular disorders in the patient’s family, but his mother did have slightly curly hair. To preliminarily determine the etiology of the patient’s diagnosis, we used the targeted next-generation sequencing (Kooshavar et al., 2018; Langabeer et al., 2018).

**FIGURE 1:**
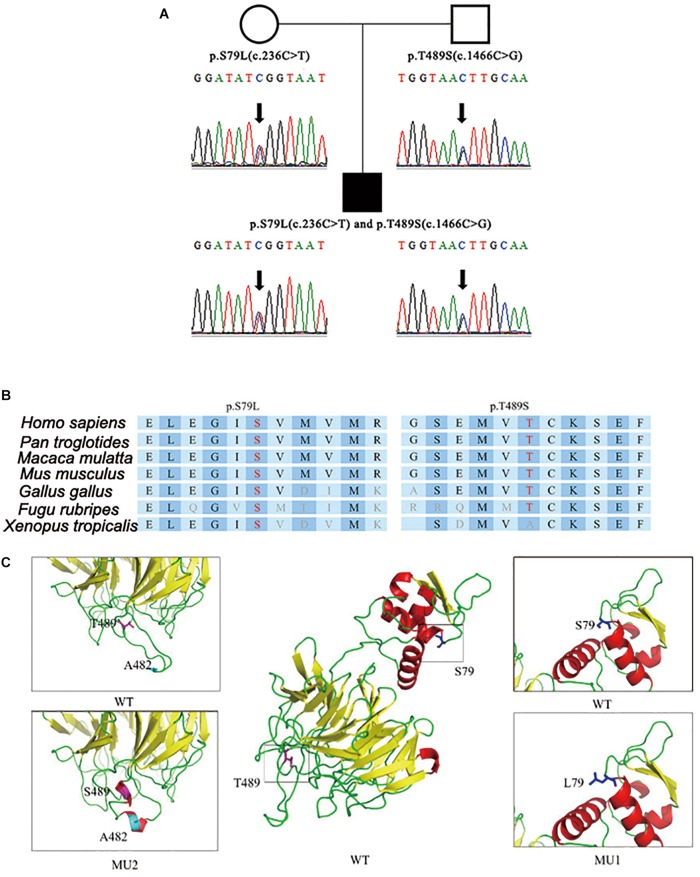
Identification of compound heterozygous mutations in the *GAN* gene. **(A)** Compound heterozygous mutations p.S79L (c.236C > T) and p.T489S (c.1466C > G) were identified in the patient’s copies of the GAN gene. **(B)** The location of the mutations with respect to the topological model of the GAN polypeptide. **(C)** Predicted crystal structures of wild-type and mutant GAN proteins. The proposed locations of the two mutations are shown in blue and pink. This model is based on template c4yy8B and covered 375 residues (32–575). The red coloring indicates helices, the yellow coloring indicates β-sheets, and the green coloring indicates loops.

After step-by-step analyses, only two compound heterozygous mutations in *GAN* survived our strict filtering process. The proband had heterozygous c.236C > T mutations in exon 2, which resulted in an amino acid substitution of serine to leucine (p.S79L) in the BTB domain (Figure 1A). Additionally, the proband had a heterozygous c.1466C > G mutation in exon 9, which resulted in a threonine-to-serine amino acid substitution (p.T489S) in the kelch domain (Figure 1A). The patient’s mother carried the c.236C > T mutation, and his father carried the c.1466C > G mutation. The c.236C > T (p.S79L) mutation was a known mutation (HGMD ID CM002979), and the c.1466C > G (p.T489S) mutation was a novel mutation. Although S79L mutation was reported (HGMD ID CM002979) here, we, for the first time, confirmed this mutation in Han Chinese. We then performed *in silico* predictions for pathogenicity for c.1466C > G mutation, which showed strong pathogenicity (Mutation Taster: disease causing; Polyphen: damaging; CADD score: 18.50). Both mutations were located in highly conserved regions of the DNA and affected the folding of the predicted protein crystal structures (Figures 1B,C).

**FIGURE 2:**
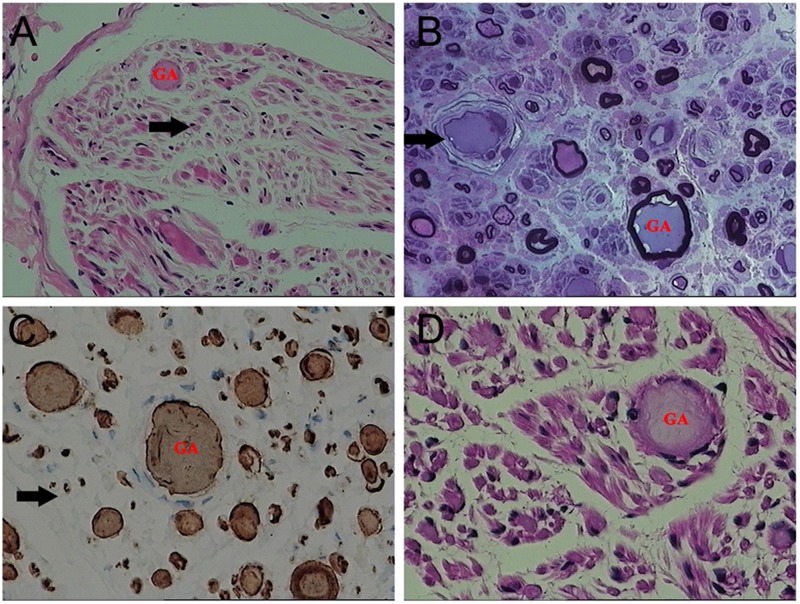
Microscopic examination of the patient’s sural nerve revealed a giant axon (GA). **(A)** HE staining revealed tumefaction of the neural axon and a few regenerating clusters (arrow). **(B)** Part of the axon atrophied mildly, and the vascular wall (arrow) was thickened and layered. **(C)** A myelinated nerve fiber (arrow) is slightly reduced. **(D)** No obvious increase in glycogen was observed.

**FIGURE 3:**
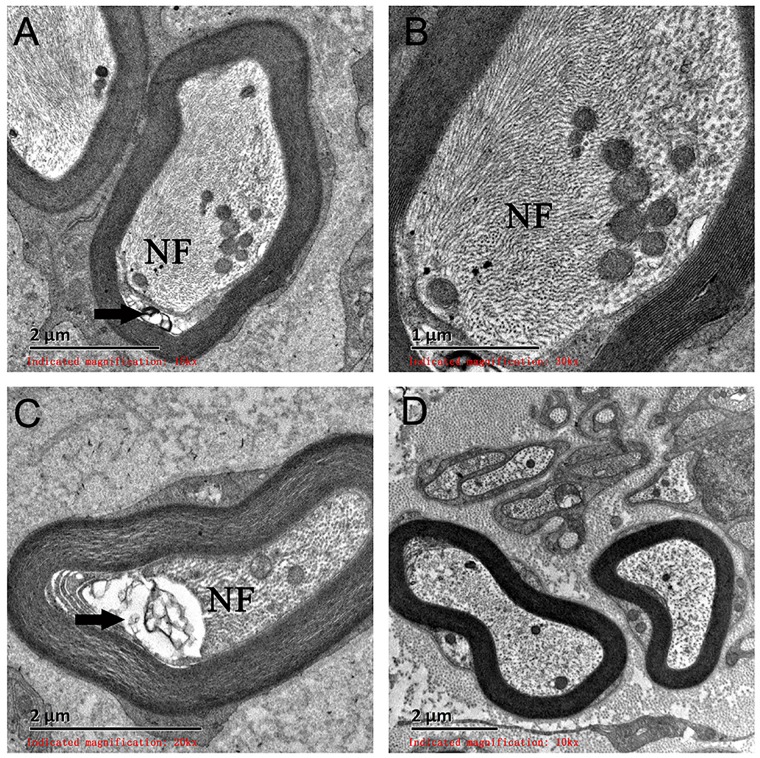
Electron microscopy examination of a giant axon (GA). **(A,B)** Electron microscopy showed giant axons filled with closely packed neurofilaments. **(C)** Neural axons were mildly atrophied (arrow) and separated from the myelin sheath. **(D)** A normal neural axon. NF, neurofilaments.

On light microscopy, parts of the nerve fibers were observed to have tumid axis-cylinder spreads, in which the neurons were depleted of their myelin sheaths (Figure 2A). Medullated fibers were mildly reduced, and thin myelinated fibers were randomly spread (Figure 2B). Immunohistochemical staining corroborated the results (Figure 2C). Tissue cells, labeled by CD163, infiltrated the nerve fibers, while lymphocytes were not present, as shown by CD8 and CD20. The PAS staining showed normal glycogen content (Figure 2D). An ultrastructural examination exposed neuronal swelling and demyelinated areas in the neural axons, which were distributed among some of the myelinated fibers (Figure 3A). However, parts of these fibers were atrophic. Giant axons were filled with closely packed and irregularly oriented neurofilaments, and the microtubules are arranged in clusters (Figure 3B). All of the affected axons exhibited thin or absent myelin sheaths (Figures 3C,D).

## Materials and Methods

DNA samples were extracted from peripheral blood samples using the QIAamp DNA Blood Midi Kit (Qiagen, Hilden, Germany). The patient’s mutation was detected by targeted next-generation sequencing, which was designed by BGI (China) and performed with the Human Sequence Capture 2.1 M Array (Roche NimbleGen, Madison, WI, United States). All of the mutations were verified by PCR and Sanger sequencing. Variant analyses were performed using an established bioinformatics pipeline as previously described (Huang et al., 2015a, b, 2017). Briefly, variants with a minor allele frequency of >0.005 in any of the variant databases were excluded. The effects of the candidate variants were assessed using *in silico* prediction programs, including Polyphen-2, MutationTaster, and CADD. We predicted the topological model using web resources, including SMART, to explore protein domain architectures and RaptorX structure prediction web server to generate both the wild-type and mutant protein models, and visualized any changes in protein folding and structure using the PyMol software. GeneMANIA was used to study the protein–protein interaction (PPI).

Sural nerve biopsy samples were obtained from the patient. First, HE staining was performed to fix one piece of the nerve in 10% formaldehyde. This piece was subsequently paraffin embedded, cut into 8-μm sections, and stained with hematoxylin and eosin. Electron microscopy was then performed on the rest of the sample, which was fixed in 2.5% glutaraldehyde and embedded in Epon 812. Semithin sections, prepared for light microscopy, were stained with toluidine blue. Ultrathin sections were contrasted with uranyl acetate and lead citrate, and then examined under an electron microscope.

## Discussion

Giant axonal neuropathy is a rare autosomal recessive neurodegenerative disease caused by mutations in the *GAN* gene. In 1972, the GAN clinical phenotype and neuropathology were first described (Asbury et al., 1972; Berg et al., 1972). The classical clinical presentation characteristics of GAN include disorder in the lower limb gait, myasthenia, ataxia, and gradual upper limb dysfunction occurring around 3 years of age. As the disease progresses, the patient becomes dysarthric and dysphagic, and ultimately passes away due to respiratory failure. In 2000, Bomont et al. reported that mutations in the *GAN* gene were the underlying cause of the GAN, and Bruno et al. subsequently confirmed this conclusion (Bomont et al., 2000; Bruno et al., 2004). The *GAN* gene encodes gigaxonin, which belongs to the BTB/kelch cytoskeleton protein family, contains an N-terminus BTB domain, a C-terminus kelch domain, and a BACK domain between the N and C terminuses. Gigaxonin has been proposed to be an E3 ligase substrate adaptor, which may be important for the turnover or regulation of intermediate filaments. Recently, Scrivo et al. demonstrated that GAN, in its capacity as an E3 ligase, regulates the ATG16L1 turnover and, in this way, controls autophagosome production and fine tunes the autophagy machinery (Scrivo et al., 2019).

Since the discovery of gigaxonin, at least 50 mutations, including missense, nonsense, insert, and splice site mutations in 11 exons of the *GAN* gene, have been reported (Johnson-Kerner et al., 2014). These mutations are scattered in the three domains and cause either the classical clinical phenotype or the mild clinical phenotype of GAN. *GAN* gene mutation detection is the accepted way to definitively diagnose GAN beyond observing the clinical presentations. We added functional analysis using protein–protein interaction (PPI) (Supplementary Figure S1) using GeneMANIA. From Supplementary Figure S1, we can see the different types of networks between related genes, suggesting that the *MAP1B*, *TBCB*, and *PRPH* genes are possibly involved in GAN. Interestingly, the role of *MAP1B* in GAN has been reported in a few studies (Ding et al., 2002; Allen et al., 2005; Ding et al., 2006).

In this report, we described a Chinese patient with GAN who had tight curly hair. The differential diagnosis of GAN was also considered. For instance, Charcot–Marie–Tooth type 2E and 1F caused by mutations in NEFL has been excluded. Electromyography showed multiple peripheral nerves that were damaged, which primarily consisted of axonal lesions and demyelination. Though the boy had the typical GAN phenotype, we could not confirm the pathogenesis without consent for the sural nerve biopsy. Instead, we employed the targeted next-generation sequencing method to test all of the known skeletal, muscle, and nervous system monogenic diseases, and used PCR to verify the mutation sites of the family. The next-generation sequencing method has proven to be a very powerful approach to identify mutations for rare diseases (Huang et al., 2018, 2019). The sequencing results showed that the patient had compound heterozygous mutations of p.S79L (c.236C > T) and p.T489S (c.1466C > G). The p.S79L mutation, reported by Bomont et al., was located in the N-terminus BTB domain of gigaxonin, which synthesizes a functional ubiquitin ligase complex with Cul3 and Rbx1, and then mediates downstream protein degradation (Bomont et al., 2000). Of note, p.S79L was previously only reported in Caucasian population. In this study, we, for the first time, confirmed this mutation in Han Chinese, indicating S79L as a common mutation in Caucasians and East Asians. However, the c.1466C > G was a novel missense mutation. This mutation resulted in amino acid substitutions of threonine to serine (p.T489S) in the C-terminus kelch domain of gigaxonin, which interacted with microtubule-associated protein 1B (MAP1B), microtubule-associated protein 8, and tubulin folding cofactor B. Similar to the other members of the BTB/kelch proteins, gigaxonin seems to interact with cytoskeletal proteins involved in various cellular processes, such as actin cytoskeleton interaction (mayven, kelch, and ENC-1 proteins), cytoplasmic sequestration of transcription factors (Keap1 protein), and cell morphology (calicin) (Ding et al., 2002, 2006). Allen et al. concluded that overexpression of gigaxonin could aggravate the degradation of MAP1B, and the ablation of gigaxonin resulted in substantial accumulation of MAP1B, both of which affect transport processes indirectly by altering microtubule dynamics (Allen et al., 2005). After we obtained consent for the sural nerve biopsy, the results from this test showed that the nerve tracts had giant axonal fibers that had been demyelinated. As a result, we were able to confirm that the GAN was the likely cause of pathogenesis.

In conclusion, compound heterozygous mutations of p.S79L (c.236C > T) and p.T489S (c.1466C > G) in the *GAN* gene were etiology of the GAN disorder in this Chinese patient. These findings assist in adding to the body of knowledge about genetic mechanisms that can result in GAN.

## Data Availability Statement

All datasets generated for this study are included in the article/Supplementary Material.

## Ethics Statement

We obtained written informed consent for genomic analysis and sural nerve biopsy of the patient and her parents in accordance with the Declaration of Helsinki. The study was approved by the Wenzhou People’s Hospital (China) Ethics Committee. The patient and his parents gave written informed consent to the publication of the information and images related to this case report.

## Author Contributions

JZ and FJ conceived the idea. XY, ZS, HZ, and HW collected the samples. XL, CZ, and CS performed the experiments. YC and WW performed the data analyses. XX wrote the manuscript. All authors have read and approved the final version of the manuscript.

## Conflict of Interest

The authors declare that the research was conducted in the absence of any commercial or financial relationships that could be construed as a potential conflict of interest.
