# Supplementary material for: Identification of Novel Compound Heterozygous Mutations in the GAN Gene of a Chinese Patient Diagnosed With Giant Axonal Neuropathy
Source: Front Neurosci. 2020 Feb 25;14:85. doi: 10.3389/fnins.2020.00085 (PMC7052293; doi:10.3389/fnins.2020.00085)
Supplement: Supplementary file 1 [file Data_Sheet_1.pdf]

frontiers

Supplementary Material

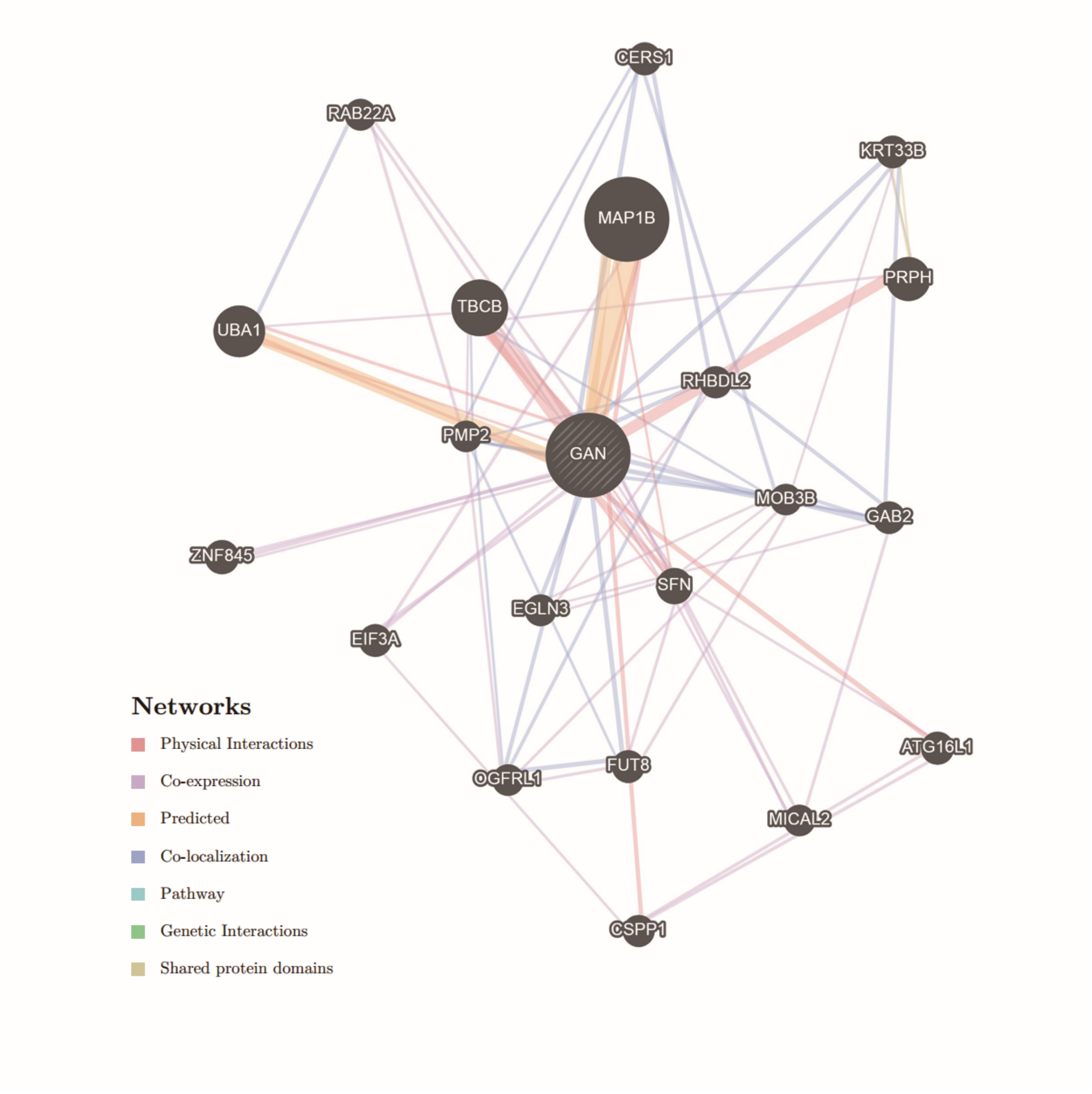

SUPPLEMENTARY FIGURE S1 | Protein–protein interaction (PPI) analysis of *GAN* gene.
